# Fall Risk Factors in the Mexican American Population: An Analysis Using the WHO Falls Risk Factor Model

**DOI:** 10.1093/geroni/igaf122.3769

**Published:** 2025-12-31

**Authors:** Hadi Kooshiar, Faezeh Babaieasl

**Affiliations:** Texas A&M University-Corpus Christi, Texas A&M University-Corpus Christi, Texas, United States; Texas A&M University-Corpus Christi, Texas A&M University-Corpus Christi, Texas, United States

## Abstract

**Background:**

Falls are a serious cause of injury, disability, and death among older adults. However, there is limited research that shows the World Health Organization (WHO) falls classification model has been applied to very old Mexican American populations. This study examined fall rates and associated risk factors among Mexican Americans aged 89 and above using the WHO model.

**Methods:**

This study uses data from Wave 10 (2020–2021) of the Hispanic Established Populations for the Epidemiologic Study of the Elderly (HEPESE).

**Results:**

The sample was composed of 66.4% females, with a mean age of 93.84 years (SD = 2.78, range = 89–103 years). 73.2% of respondents had mild cognitive impairment. The fall rate was 39.7%, with 20% of participants experiencing multiple falls, and 16.8% of falls leading to injury. Additionally, 74.3% of respondents reported a fear of falling. According to the WHO falls risk model, in the biological domain, fallers scored lower on Instrumental Activities of Daily Living (IADL) and Activities of Daily Living (ADL), had higher depression rates, and more often had dementia. In the socioeconomic domain, marital status differed significantly between fallers and non-fallers. In the environmental domain, homeownership was significantly associated with fall status.

**Conclusion:**

Applying the WHO fall risk factor model revealed multiple areas of vulnerability in very old Mexican Americans. Cognitive and functional impairments, environmental factors, and marital status are significant factors that increase the risk of falls. These findings underscore the importance of culturally tailored, comprehensive fall prevention programs for this aging population.

